# THRIL mediates endothelial progenitor cells autophagy via AKT pathway and FUS

**DOI:** 10.1186/s10020-020-00201-2

**Published:** 2020-09-09

**Authors:** Jiandong Xiao, Yuli Lu, Xinchun Yang

**Affiliations:** 1 Department of Cardiology, Beijing Chaoyang Hospital, Capital Medical University, No.8 Gongren Tiyuchang Nanlu, Chaoyang District, Beijing, 100020 China; 2 Department of Cardiology, Hengshui People’s Hospital, Hengshui, 053400 Hebei Province China; 3 Department of Endocrine, Hengshui People’s Hospital, Hengshui, 053400 Hebei Province China

**Keywords:** THRIL, Coronary heart disease, AKT, FUS

## Abstract

**Background:**

This study focused on the roles of lncRNA THRIL in coronary atherosclerotic heart disease (CAD) through regulating AKT signaling pathway and directly interacting with FUS.

**Methods:**

QRT-PCR was conducted to detect the expression of THRIL in CAD blood samples and endothelial progenitor cells (EPCs). Cell autophagy of EPCs was examined through Cyto-ID Autophagy Detection Kit. CCK-8 assay and flow cytometry were carried out to assess cell viability and apoptosis under various interference conditions. Western blotting was conducted to detect the expression of interest proteins. The expression levels of vascular cell adhesion molecule-1 (VCAM-1) and intercellular adhesion molecule-1 (ICAM-1) were measured by qRT-PCR. The direct interactions between HCG18 and FUS was confirmed through RNA electrophoretic mobility shift assay (RNA EMSA) and RNA immunoprecipitation (RIP) assay.

**Results:**

THRIL was upregulated in CAD blood samples and EPCs. Knockdown of THRIL in EPCs promoted cell viability, inhibited cell autophagy and further suppressed the development of CAD. Over-expression of THRIL induced inactivation of AKT pathway, while knockdown of THRIL played reversed effects. THRIL directly interacted with FUS protein and knockdown of FUS reversed the over-expressing effect of THRIL on cell proliferation, autophagy and the status of AKT pathway.

**Conclusion:**

THRIL inhibits the proliferation and mediates autophagy of endothelial progenitor cells via AKT pathway and FUS.

## Introduction

Coronary atherosclerotic heart disease (CAD) is one of the chronic diseases with very high mortality rate due to the acceleration of population aging process (Corral-Debrinski et al., 1992). The level of risk factors for cardiovascular disease (CVD) continues to increase all over the world (Becker, 1985). Moreover, the incidence of CAD and the resulting social and economic burden are increasing (Ornish et al., 1990). Although cruuent treatments such as medical treatment, percutaneous coronary intervention (PCI), and coronary artery bypass surgery have improved the prognosis of CAD, the mortality rate remains high (Keys, 1975). It is urgent to explore the pathological mechanism of CAD, optimize the treatment strategy, and enable early diagnosis of CAD. Recently, autophagy has become a new research interest with the increased understanding of the pathogenesis of atherosclerosis. It has been reported that autophagy is closely related to cancer, neurodegenerative diseases, and CVD (Rebecca & Amaravadi, 2016; Deng et al., 2017). Autophagy is a process related to lysosomal proteolytic mechanism and it can remove harmful proteins from cells to maintain a healthy state under stress conditions (Boya et al., 2013). Studies have shown that cell autophagy impairs atherosclerosis process while defective autophagy in cells enhances atherosclerosis (Xiong et al., 2014; Osonoi et al., 2018). Progenitor (stem) cells are immune system cells characterized by the ability to self-renew and differentiate into various cell types (Xu, 2008). Endothelial progenitor cells (EPC) differentiate into endothelial cells (EC) and participate in endothelium recovery, new blood vessels formation, and suppression of atherosclerosis (Zhang et al., 2014). Therefore, EPCs have been used as an important cell model to investigate gene function or drug effect in CAD (Zhu et al., 2019; Ansheles et al., 2017).

Non-coding RNAs have been reported as new biomarkers for diagnosis in the field of oncology and new targets for cancer treatment (Yang et al., 2014). However, understanding of the role of CVD is still of great importance. Long noncoding RNAs (lncRNAs, > 200 nt) have been shown to be involved in the pathophysiological processes of coronary artery disease (Greco et al., 2016). One study reported that increased expression levels of lncRNA H19 in peripheral blood mononuclear cells were related with risk of coronary artery disease (Bitarafan et al., 2019). LncRNA THRIL (TNFα and hnRNPL related immunoregulatory lincRNA) is firstly reported to modulate the expression of TNFα through the interactions with hnRNPL, and plays an important role in regulating inflammation response (Xia et al., 2019). In addition, knockdown of lncRNA THRIL could protect hypoxia-induced injury of H9C2 cells through regulating miR-99a (Xia et al., 2019). However, the functions of THRIL in CAD are rarely reported.

AKT is a downstream signaling pathway of receptor tyrosine kinase and an important pathway for membrane receptor signaling to be transmitted intracellularly (Chaanine & Hajjar, 2011). It plays an important regulatory role in biological processes such as embryonic development, cell differentiation, proliferation, and death (Hers et al., 2011). Animal model studies have shown that AKT is an important regulator of cardiac development (Fujio et al., 2000). AKT mutant mice have abnormal cardiovascular system phenotypes (Kumarswamy et al., 2012). As an RNA/DNA binding protein, FUS is involved in mRNA transcription, splicing, transport and maturation (Marrone et al., 2018; Fiesel & Kahle, 2011). Besides, previous studies have reported that AKT and FUS participate in cell autophagy, which could pose protective effects in the progression of CAD (Ryu et al., 2014; Ling et al., 2019). Therefore, RNAs which were abnormally metabolized caused by mutation of FUS may play a key role in the occurrence of CAD, but the current understanding of its pathogenesis is still limited. Here in this study, the role and interactions of lncRNA THRIL, AKT pathway and FUS in the development of coronary heart disease were investigated.

## Methods

### Samples

20 CAD patients and 20 healthy volunteers were enrolled in Beijing Chaoyang Hospital from October 2017 to December 2019. We obtained their atherosclerotic peripheral blood as samples. The total cholesterol, low-density lipoprotein (LDL) cholesterol, triglycerides from the patients were tested. All the participants signed the informed consents. This study was approved by the Ethics Committee of Beijing Chaoyang Hospital. Peripheral blood was centrifuged at 1000 g at 4 °C for 10 min. Plasma samples were centrifuged it at 16,000 g at 4 °C for 10 min. The supernatant was stored at − 80 °C.

### Endothelial progenitor cell isolation and culture

First, 2 ml peripheral blood was collected from each patient with CAD (*n* = 20) and healthy donors (*n* = 20). Through ficoll density gradient centrifugation, the peripheral blood mononuclear cells were isolated. These cells were then cultured on six-well plates coated with fibronectin for 24 h before transplanting, and in the endothelial basal medium (Cambrex, Walkersville, MD, USA) supplemented with human recombinant long insulin-like growth factor-1, ascorbic acid, vascular epidermal growth factor (Preprotech, Rocky Hill, NJ, USA), cortisol and 20% FBS (Hyclone, South Logan, UT, USA) at 37 °C in a 5% CO2 incubator. To screen the EPCs, non-adherent cells were removed by washing with PBS after 4 d, and the adherent cells (attached early EPCs) were incubated in fresh medium every 3 d. The morphological characteristic of EPCs was elongated spindle-shape. To further identify their phenotype, flow cytometry analysis (Becton Dickinson, Franklin Lakes, NJ, USA) was conducted to assess the surface markers. FITC-conjugated antibodies of CD31, CD34 and CD45 (Abcam, Cambridge, MA, USA) were used (Ikutomi et al., 2015).

### Cell transfection

The transfection sequences were designed by Sangon, China. Negative control (NC), shTHRIL-1, shTHRIL-2, pCMV6-THRIL, NC-inh and FUS-inh were transfected through Lipofectamine 3000. The transfected cells were collected after 48 h. Normal: EPCs from healthy controls; NC: EPCs isolated from CAD patients. All the transfection experiments were performed in EPCs isolated from CAD patients.

### qRT-PCR

Total RNAs were extracted from EPCs using TRIzol (Invitrogen, Carlsbad, CA, USA) following the manufacturer’s instructions, and cDNA was reverse transcribed from RNA using SuperScript III (Invitrogen). The expression of THRIL, VCAM-1 and ICAM-1 were evaluated by the SYBR green quantitative PCR kit (Takara, Tokyo, Japan). β-actin was used as an internal control. Gene expression levels were calculated by 2^-ΔΔCt^ method. The primers used in this study were as follows:

THRIL-F: 5′-AGGTCTGGCAGGGGTTATCT-3′.

THRIL-R: 5′-TGGGGATCACGACTGTCTCT-3′.

VCAM-1-F: 5′-GGACCACATCTACGCTGACA-3′.

VCAM-1-R: 5′-TTGACTGTGATCGGCTTCCC-3′.

ICAM-1-F: 5′-TCTTCCTCGGCCTTCCCATA-3′.

ICAM-1-R: 5′-AGGTACCATGGCCCCAAATG-3′.

β-actin-F: 5′-GCATGGGTCAGAAGGATTCCT-3′.

β-actin-R: 5′-TCGTCCCAGTTGGTGACGAT-3′.

### Cell counting kit-8 assay

Cell proliferation was measured by cell counting kit-8 (CCK-8) (Dojindo, Kumamoto, Japan). Cells were seeded in 96-well plates at 5 × 10^3^ cells per well and subjected to the aforementioned treatments. Then, 10 μL of CCK-8 solution was added into each well and incubated for an additional 2 h. The optical density value at 450 nm was measured using a microplate reader (Autobio Diagnostics Co, Ltd., Zhengzhou, China).

### Flow cytometry

After washing in ice-cold PBS, cells in 10 M MHEPES/NaOH were re-suspended in Annexin V binding buffer. Then 5 ul FITC-Annexin V was added into EPCs suspension. After propidium iodide staining, the cells were analyzed through flow cytometry (CytoFLEX LX, Beckman).

### Cell autophagy analysis

EPCs autophagy was evaluated by Cyto-ID Autophagy (Enzo, USA). LC3II-positive punctate pattern was observed through confocal microscope. Numbers of autophagosomes were measured using ImageJ software.

### Western blot

Cellular extracts were lysed using the lysis buffer RIPA, which was purchased from KeyGen Biotech Co. Ltd. (Nanjing, China), and the supernatant was collected after centrifugation. Proteins were separated using sodium dodecyl sulfate-polyacrylamide gel electrophoresis (SDS-PAGE) and were blotted onto polyvinylidene difluoride membranes (Bio-Rad, USA). Then, membranes with isolated proteins were blocked for 1 h and incubated with primary antibodies including anti-mTOR (ab2732, 1:2000), anti-mTORC1 (ab137133, 1:1000), anti-ATG1 (ab167139, 1 μg/ml), anti-LC3-II (ab48394, 1 μg/ml), anti-β-actin (ab8227, 1:100), anti-AKT (ab64148, 1:1000) and anti-p-AKT (ab8933, 1:1000) at 4 °C overnight. After that, membranes were washed and treated with HRP-Anti-Rabbit IgG H&L (1:2000) for 1 h. Chemiluminescence was used to quantify the blots.

### RNA Electrophoretic Mobility Shift Assay (EMSA)

RNA EMSA was conducted using EMSA Kit (Thermo Fisher, USA) with nuclear extract of EPCs. Then 10 pM labeled probes were incubated with proteins in REMSA binding buffer with 2 μg tRNA and 5% glycerol at 25 °C for 30 min. Unlabeled probes (1 μM) were used for competitions. The reaction was loaded to 6% polyacrylamide and sent to nylon membranes (Roche, USA). Next, they had cross-linking with the membranes by UV-light. HRP-conjugated streptavidin was used to quantify the results, along with ECL.

### RNA Immunoprecipitation (RIP)

Briefly, cells were firstly cross-linked and lysed. Nuclei was isolated and incubated with primary antibodies at 4 °C overnight with Magnetic Beads (Millipore, Germany). The beads were washed and re-suspended in RIPA with Proteinase K and incubated for 1 h. TRIzol was used for RNA extractions. Anti-FUS (Abcam, 1:200) was used for RIP. Total RNAs and IgG were assayed simultaneously.

### Statistical analysis

Student’s t-test and one-way ANOVA were utilized to analyze the difference between 2 groups or among multiple groups, respectively. Two-sided *P* values less than 0.05 were regarded as statistical significance. All data followed normal distributions and were presented as mean ± stand deviation (SD).

## Results

### LncRNA THRIL was up-regulated in CAD blood samples and inhibits the proliferation of EPCs

**Fig. 1:**
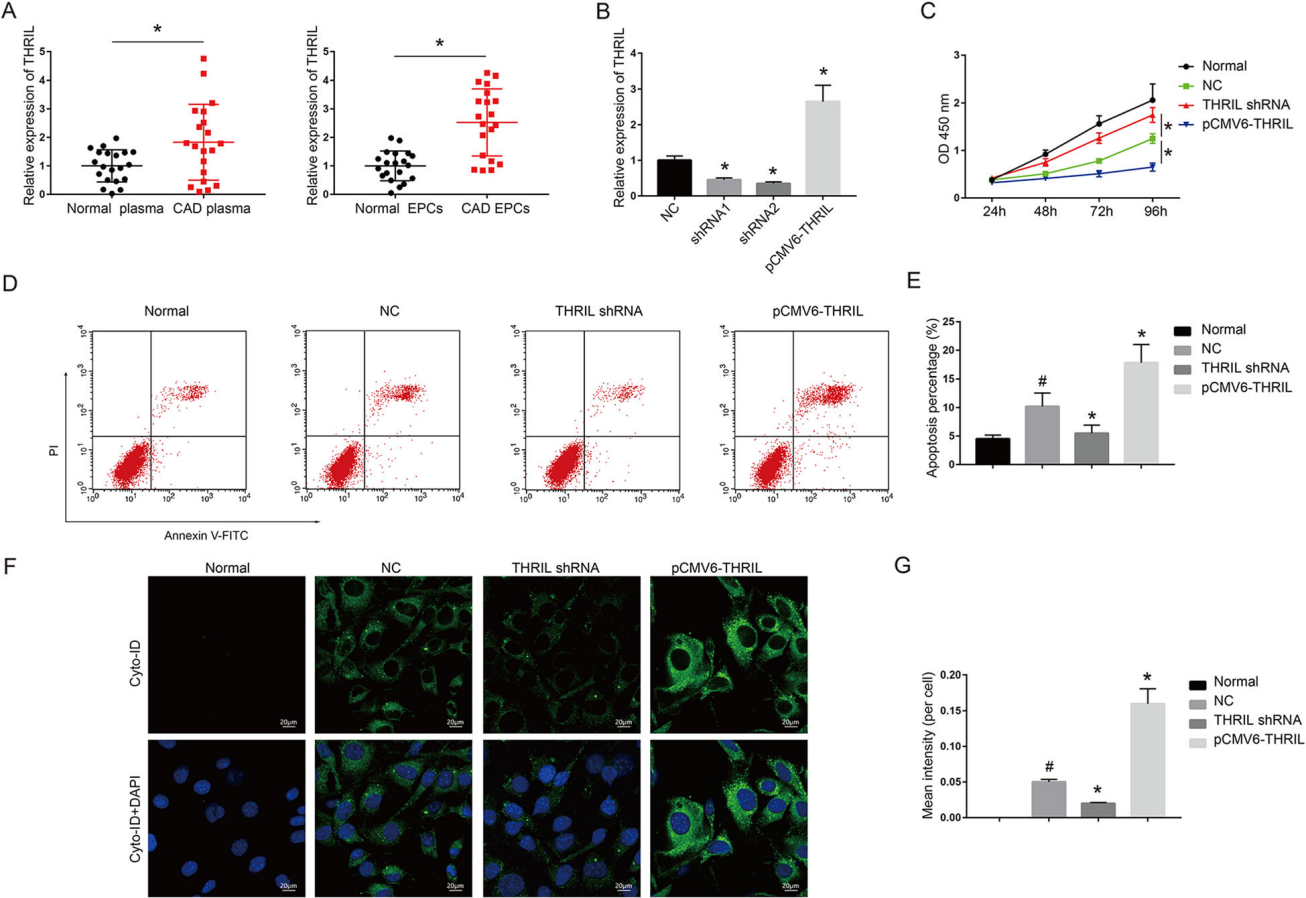
LncRNA THRIL induced cell autophagy and promotes CAD progression. (**a**) THRIL was overexpressed in CAD blood samples and EPCs. *P < 0.05, compared with normal (healthy) group. The number of CAD patients and healthy controls were all twenty. (**c**) THRIL was depressed in EPCs in transfection with sh-THRIL-1 or sh-THRIL-2 and overexpressed through using pCMV6-THRIL. The RNA expression of THRIL was measured through qRT-PCR. *P < 0.05, compared with NC group. (**c**) Cell viability was measured through using CCK-8 assays. (**d**) Flow cytometry results revealed that sh-THRIL reduced apoptotic cell number of EPCs. (**f**) Autophagy assays results revealed that sh-THRIL reduced EPCs autophagy rate. Normal: EPCs from healthy controls; NC: EPCs isolated from CAD patients; All the transfection experiments were performed in EPCs isolated from CAD patients. *P < 0.05, compared with NC group; #*P* < 0.01, compared with normal ones. The data shown represent three separate experiments performed in triplicate in each experiment (mean ± the standard deviation [SD])

To detect the expression of THRIL, qRT-PCR was conducted and the results showed that the expression levels of THRIL were greatly enhanced in CAD blood and EPC (*P* < 0.01, Fig. 1a). EPCs in transfection with either shRNA1 or shRNA2 showed suppressed expression in contrast to NC while pCMV6-THRIL was enhanced (*P* < 0.01, Fig. 1b). And the expression levels of sh-THRIL-2 were even lower than that in sh-THRIL-1 group. shRNA2 was therefore used for the following experiments. CCK-8 assay revealed that THRIL shRNA promoted cell viability of EPCs in CAD and pCMV6-THRIL obviously inhibited cell viability compared with that in NC (*P* < 0.01, Fig. 1c). However, no difference was found in healthy EPCs and NC in cell viability (*P* < 0.01, Fig. 1c). Flow cytometry assay verified that apoptotic cell number in NC was elevated compared with normal ones (*P* < 0.05, in Fig. 1d and e). Furthermore, THRIL shRNA had lower apoptotic cell number in EPCs (*P* < 0.01, Fig. 1d and e). The apoptotic cell number in pCMV6-THRIL was elevated compared with that in NC (*P* < 0.05, Fig. 1e). Autophagy assay demonstrated that there was almost no autophagy in normal cells. pCMV6-THRIL promoted cell autophagy in EPCs compared to NC and THRIL shRNA had an inhibitory effect (*P* < 0.05, *P* < 0.01, Fig. 1f).

### LncRNA THRIL inhibits AKT signaling pathway and the expression of ATG1/LC3-II

**Fig. 2:**
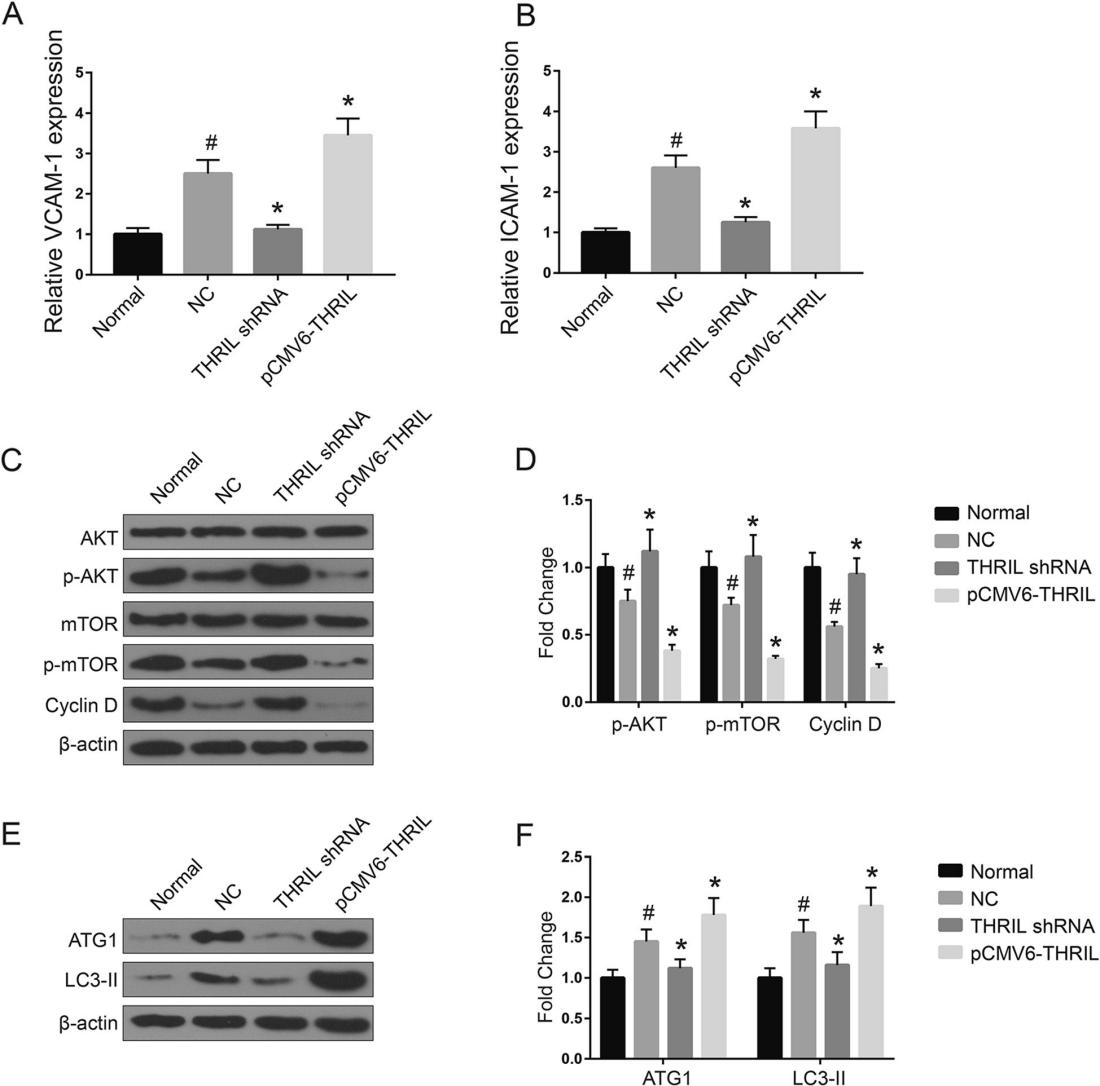
The expression of VCAM-1/ICAM-1 and AKT signaling pathway in cells. (**a**) and (**b**) QRT-PCR results demonstrated that shTHRIL suppressed the expression of VCAM-1 and ICAM-1 and over-expression of THRIL could promote CAD progress. (**c**) and (**d**) AKT signaling pathway expressions in various transfection groups. (**d**) The expressions levels of ATG1 and LC3-II in various transfection groups. Normal: EPCs from healthy controls; NC: EPCs isolated from CAD patients; All the transfection experiments were performed in EPCs isolated from CAD patients. *P < 0.05, compared with NC group; #*P* < 0.05, compared with normal ones. The data shown represent three separate experiments performed in triplicate in each experiment (mean ± the standard deviation [SD])

To further determine whether THRIL was involved in the regulation of AKT pathway, we firstly measured the expression of AKT under various situations by western blotting. Western blotting results demonstrated that the expression of p-AKT, p-mTOR and Cyclin D1 were down-regulated through pCMV6-THRIL while they were promoted through THRIL shRNA. The expression of CAD markers, VCAM-1 and ICAM-1, were measured by qRT-PCR. The results suggested that the expression levels of VCAM-1 and ICAM-1 in NC were markedly elevated compared with that in normal samples (*P* < 0.01, Fig. 2a and b). Besides, THRIL shRNA could significantly down-regulate the expression of VCAM-1/ICAM-1 (*P* < 0.01, Fig. 2a and b). The expression levels of the three signal proteins in NC were significantly lower than that in normal ones (*P* < 0.05, Fig. 2c and d). Moreover, autophagy positive markers LC3-II and ATG1 were highly expressed in pCMV6-THRIL while downregulated in THRIL shRNA (*P* < 0.05, *P* < 0.01, Fig. 2e and f). Taken together, these data indicated that lncRNA THRIL promotes CAD progression through regulation of cell proliferation and autophagy.

### THRIL promoted apoptosis through binding with FUS

**Fig. 3:**
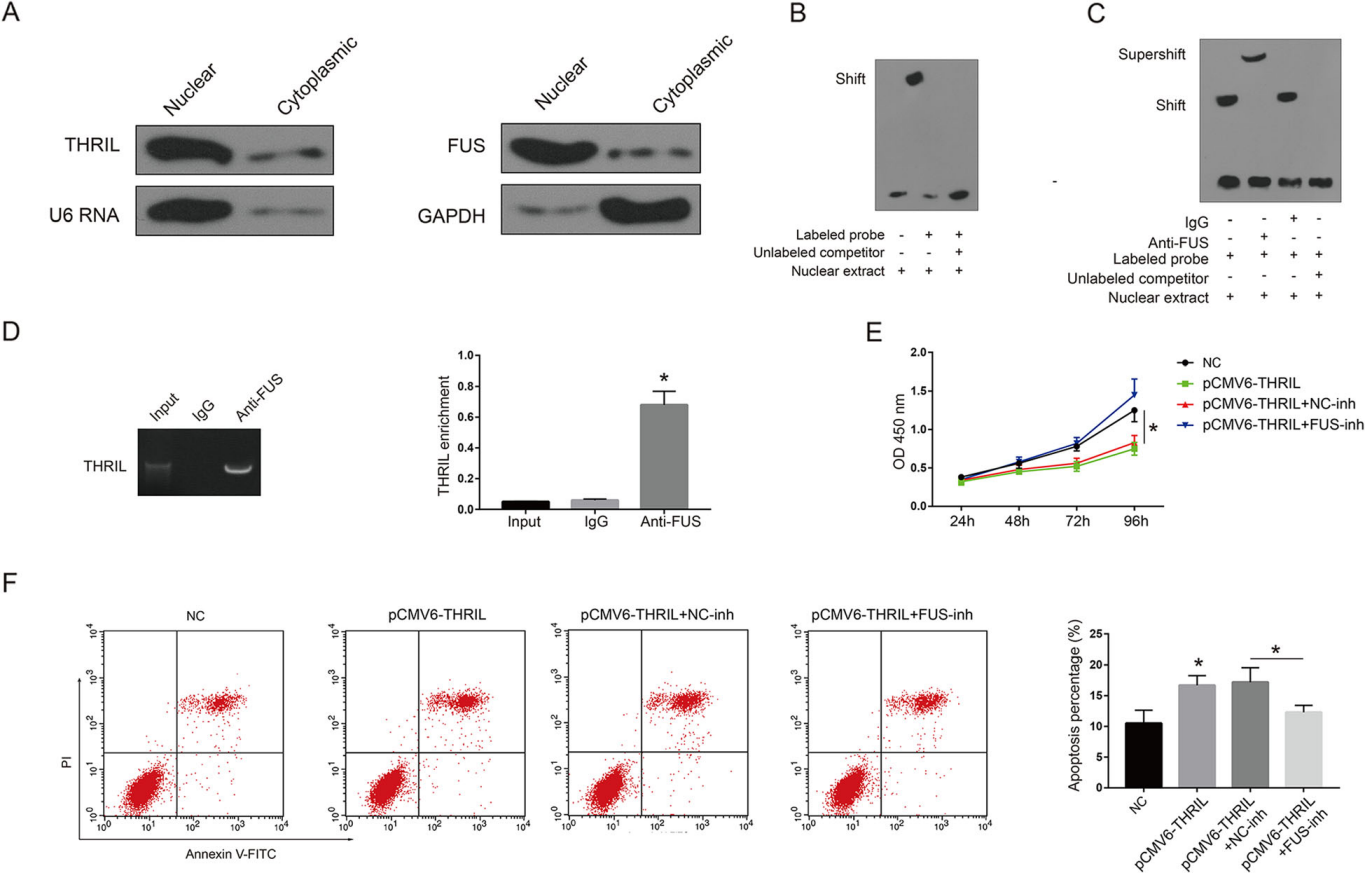
THRIL inhibits proliferation and promoted apoptosis through binding with FUS (**a** left) The expression of THRIL in CAD EPCs cell nucleus and cytoplasm was measured through northern blotting. (**a** right) The expression of FUS in CAD EPCs cell nucleus and cytoplasm was measured through western blotting. (**b**-**d**) The direct interactions between THRIL and FUS was confirmed through RNA electrophoretic mobility shift assays (RNA EMSA) and RNA immunoprecipitation (RIP) assays. (**e**) The effects of THRIL over-expression on cell viability of CAD EPCs was reversed through knockdown of FUS. (**f**) The effects of over-expression of THRIL on cell apoptosis of CAD EPCs was reversed through knockdown of FUS. NC: EPCs isolated from CAD patients; All the transfection experiments were performed in EPCs isolated from CAD patients. Data are presented as mean ± SD. *P < 0.05. The data shown represent three separate experiments performed in triplicate in each experiment (mean ± the standard deviation [SD])

As shown in Fig. 3a, western blotting indicated that THRIL and FUS were both located in cell nuclear. Through EMSA, we found FUS could bind with THRIL probe efficiently (Fig. 3b). In addition, supershift assays revealed that the band of RNA–protein complex was successfully shifted with the anti-FUS antibodies in (Fig. 3c), which confirmed the interactions between THRIL and FUS. A great enrichment of THRIL in immunoprecipitates with FUS was observed, but not with IgG, suggesting the presence of THRIL–FUS complex (Fig. 3d). In addition, knockdown of Fus through lentivirus-Fus-sh (shRNA) could reverse the effects of pCMV6-THRIL on EPCs proliferation and reduce the percentage of apoptotic cells induced through over-expression of THRIL in CAD EPCs (Fig. 3e-g).

### The effects of THRIL on AKT pathway and autophagy pathway were reversed through knockdown of FUS

**Fig. 4:**
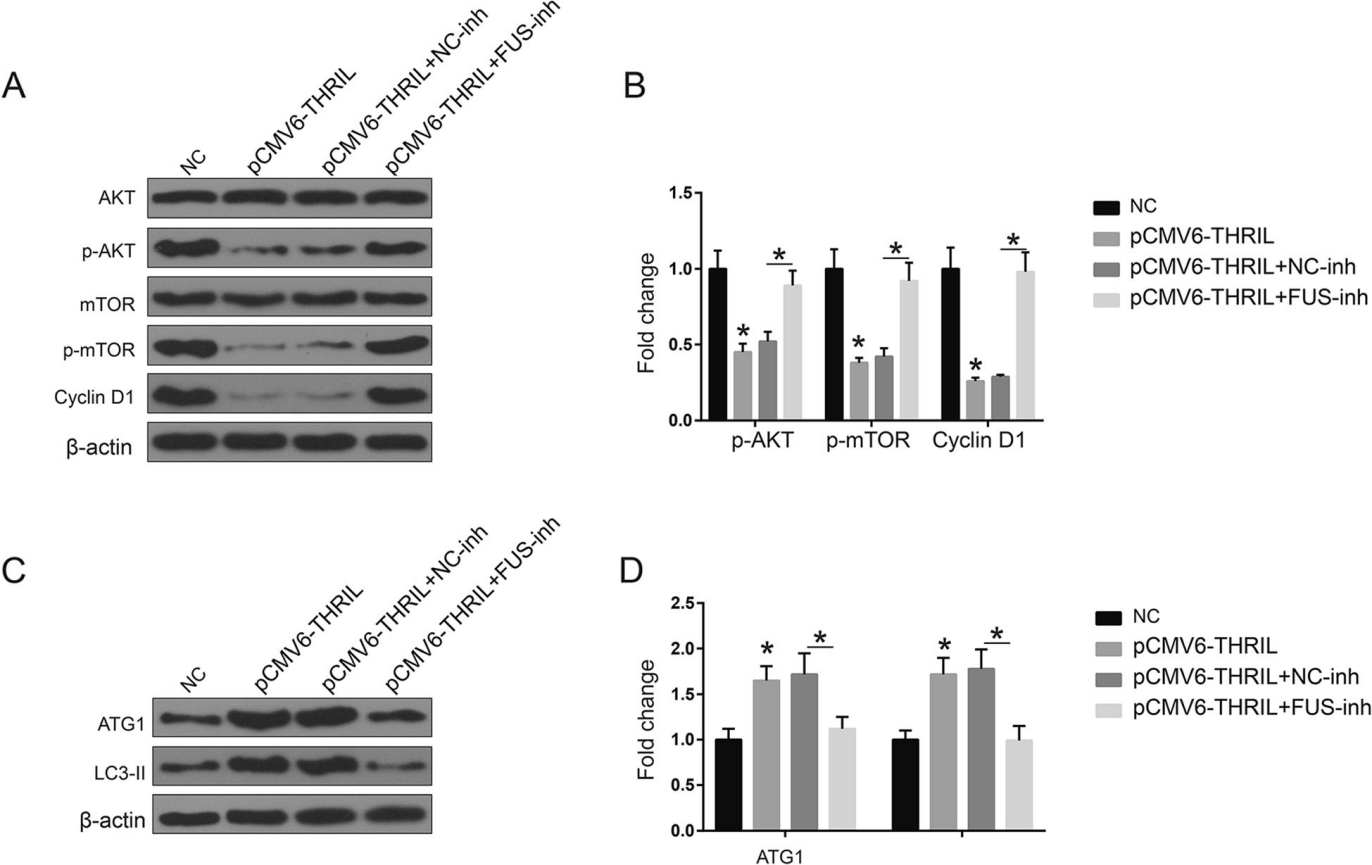
The effects of THRIL on AKT pathway and autophagy pathway were reversed through knockdown of FUS. (**a**) and (**b**) AKT signaling pathway expressions in various transfection groups. (**c**) and (**d**) ATG1 and LC3-II expression levels in various transfection groups. NC: EPCs isolated from CAD patients; All the transfection experiments were performed in EPCs isolated from CAD patients. *P < 0.05, compared with NC group. The data shown represent three separate experiments performed in triplicate in each experiment (mean ± the standard deviation [SD])

Moreover, we also investigated the role of FUS in the regulation of AKT and autophagy pathways induced through THRIL. The results indicated that FUS-inh could reverse the effects of pCMV6-THRIL on the expression of AKT pathway and ATG1/LC3-II (Fig. 4 a-d). Overall, the results indicated that THRIL could promote CAD progression via direct binding with FUS protein.

## Discussions

Extensive efforts have been made in the discovery of lncRNAs in CAD. For instance, it was reported that the expression levels of lncRNA H19 and LIPCAR were increased and associated with increased risk of CAD in a Chinese population (Zhang et al., 2017). Another study also reported the association of polymorphisms in MALAT1 with risk of coronary atherosclerotic heart disease in a Chinese population (Wang et al., 2018). From our experiments, the qRT-PCR showed that the expression of THRIL in CAD blood samples and EPCs were significantly enhanced. EPCs in transfection with either shRNA1 or shRNA2 demonstrated lower expression levels in contrast to NC group, but pCMV6-THRIL had higher expressions levels. CCK-8 assay revealed that THRIL shRNA promoted cell viability of CAD EPCs and pCMV6-THRIL inhibited cell viability compared with NC obviously. Flow cytometry assay found that apoptotic cell number in NC was elevated compared with that in normal ones. The apoptotic cell number in pCMV6-THRIL was elevated compared with that in NC group. Autophagy assay demonstrated no autophagy in normal ones. For the first time, we report that the expression of lncRNA THRIL was upregulated in the blood samples of CAD, and THRIL could promote CAD progression.

LncRNA ANRIL was illustrated to be involved in the inflammation-relevant CAD, in which the expression levels of inflammatory factors including IL-6, IL-8, NF-κB, TNF-α, iNOS, ICAM-1, and VCAM-1 were elevated in CAD mice compared to that in the control group (Guo et al., 2018). Our data suggested that the transcriptional levels of VCAM-1 and ICAM-1 in NC were elevated compared with that in normal ones. Besides, THRIL shRNA could significantly down-regulate the expression of VCAM-1/ICAM-1. In addition, previous studies suggested that autophagy process could alter differentiation potency of CD146^+^ cells into mature pericyte, endothelial, and cardiomyocyte lineage, which was related to cardiac regeneration (Hassanpour et al., 2020; Hassanpour et al., 2019). This finding is in consistence with the previous studies.

AKT signaling pathway was reported to be hypoactivated by synergistic actions of diabetes mellitus and hypercholesterolemia resulting in advanced coronary artery disease (Hamamdzic et al., 2010). Accumulative studies also suggested that AKT signaling pathway participates closely in CAD progression (Erdogdu et al., 2010). Western blotting assay demonstrated that the expression of p-AKT, p-mTOR and Cyclin D1 were down-regulated through pCMV6-THRIL while they were promoted through THRIL shRNA. The expression of the three signal proteins in NC was significantly lower than that in normal ones. LC3-II was over-expressed in pCMV6-THRIL while down-regulated in THRIL shRNA group. In agreement with previous findings, our results confirmed that lncRNA THRIL inhibits AKT signaling pathway and the expression of ATG1/LC3-II.

Autophagy of cardiomyocytes can protect cells and reduce cell loss, while autophagy can also cause cardiomyocyte death (Nishida et al., 2009). As far as we know, the role of FUS in the progression of CAD has not been reported before. However, FUS protein is closely involved in the cell autophagy. For example, one study investigating the functions of FUS and autophagy in neurodegenerative diseases revealed that FUS could mediate the cell autophagy during the progression (Kiriyama & Nochi, 2015). Herein, we also investigated the role of FUS protein in the regulation of AKT and autophagy pathways induced through THRIL. Our data indicated that FUS-inh could reversed the effects of pCMV6-THRIL on the expression of AKT pathway and ATG1/LC3-II. For the first time, we indicated that THRIL could promote CAD progression via direct binding with FUS protein.

## Conclusion

THRIL inhibits the proliferation and mediates autophagy of endothelial progenitor cells via AKT pathway and FUS protein.

## Data Availability

The analyzed data sets generated during the study are available from the corresponding author on reasonable request.

## Abbreviations

CAD: Coronary atherosclerotic heart disease
VCAM-1: Vascular cell adhesion molecule-1
EPCs: Endothelial progenitor cells
ICAM-1: Intercellular adhesion molecule-1
RNA EMSA: RNA electrophoretic mobility shift assays
RIP: RNA immunoprecipitation
